# Repeatability and reproducibility of a hyperspectral imaging system for in vivo color evaluation

**DOI:** 10.1111/srt.13160

**Published:** 2022-05-23

**Authors:** Carl Blaksley, Kumiko Udodaira, Mie Yoshida, Alexandre Nicolas, Damien Velleman, Marco Casolino, Frederic Flament

**Affiliations:** ^1^ L'Oréal Research and Innovation Kawasaki Japan; ^2^ RIKEN Wako Japan; ^3^ Istituto Nazionale di Fisica Nucleare Sezione di Roma Tor Vergata Rome Italy; ^4^ Dipartimento di Fisica Universitá degli Studi di Roma Tor Vergata Rome Italy; ^5^ L'Oréal Research and Innovation Chevilly‐Larue France

**Keywords:** color evaluation, hyperspectral imaging, instrumental uncertainty, repeatability, reproducibility, spectral imaging, systematic uncertainty

## Abstract

**Background:**

Color imaging is a tried and true method for the evaluation of cosmetic and dermatological effects, but it fails to capture all the information in a scene's spectral reflectance. For this reason, there has been in recent years increasing interest in the use of imaging spectrometers for clinical studies and product evaluation.

**Material and methods:**

We developed a novel HyperSpectral Imager (HSI) able to take in vivo full‐face format images as a next generation instrument for skin color measurement and beyond. Here, we report part of the results of our first full‐scale validation test of the HSI. We replicated a make‐up foundation screening test by applying three products to a panel of 9 models and evaluated the product *L* ^∗^, *a* ^∗^, *b* ^∗^, and ∆*E* effect immediately after application relative to the bare skin condition. We repeated this test twice in order to study the repeatability of the HSI as an evaluation instrument and during each test two different operators duplicated the data acquisition so we can assess the reproducibility of the measurements.

**Results:**

We find that the measurements from the HSI provide repeatability and reproducibility as good or better than those of our previous benchmark devices.

**Conclusion:**

From these results, we conclude that not only is the HSI suitable for use in color evaluation studies, but also that it gives operational advantages over the previous generation of evaluation instruments, as it provides a spectral measurement *combined* with good spatial resolution. This allows for analysis of color over an area and *post hoc* selection of study regions and so opens new possibilities for studies of complex in vivo phenomena which neither non‐imaging spectrometers nor conventional cameras can pursue. This study also raises points for future work concerning proper inclusion of instrument uncertainty in comparisons of results between instruments and handling of systematic uncertainties from analyses based on a single area.

## 1 INTRODUCTION

The field of cosmetics is by definition a visual medium, and visual assessments, whether in the form of self‐judgment, expert evaluation, or instrumental measurements are one of the key components of both the actual and perceived efficacy of cosmetic products. At the same time, the limitations of color imaging as an instrumental measurement of appearance are well known. Among these are metamerism effects and color change under different illuminations. These issues have long been known in industries such as printing, pigment manufacturing, and textiles, where accurate color matching is fundamental. Beyond simple color matching, however, it is important to remember that RGB color imaging condenses all the spectral information in a scene into three band passes, and therefore fails to capture the complete picture which an object presents. To overcome these limitations, we can instead measure the reflectance or emission spectrum of an object, and we can put the measured spectra in different spectral bands to uses such as stellar composition analysis in astronomy,^1^, ^2^ geological surveys from the air or from space,^3^, ^4^, ^5^ and, closer to the field of skin research, tracking blood oxygenation and flow,^6^, ^7^ mapping melanin content in skin,^8^, ^9^, ^10^ or diagnosing skin lesions as benign or cancerous.^11^, ^12^

Considering these possibilities, there has been increasing interest in the use of multispectral and hyperspectral imaging for use in clinical, research, and evaluation settings in recent years, and in that context we have worked to update the *Chromasphere* system,^13^ our reference device for skin color measurement, by replacing the RGB cameras with an imaging spectrometer. For this application, the imaging spectrometer must provide a spatial resolution on par with the previous color cameras and a spectral resolution near that of a spectroradiometer, while also achieving a time resolution suitable for in vivo studies of the full human face. Due to this combination of requirements, spatial scanning spectrometers are not suitable, and so we developed a new HyperSpectral Imager (HSI) based on a tunable liquid‐crystal birefringent filter.^14^

The HSI measures the reflectance spectrum in each pixel of the field of view, and thus provides a “color” measurement which is independent of the illumination. At the same time, the spatial resolution of the HSI allows for both *post hoc* selection of multiple regions of interest (ROI) as well as for analyses of the spatial variance of the reflectance spectrum. In this paper, we report an assessment of the repeatability and reproducibility of the HSI measurements, as these are key aspects of any evaluation platform.

## 2 MATERIALS AND METHODS

**FIGURE 1:**
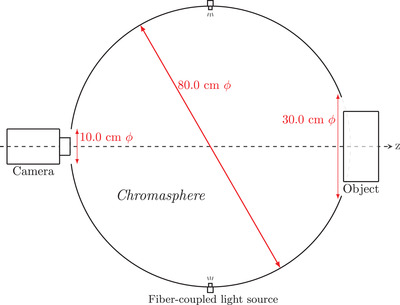
Overview of the *Chromasphere* system. A light source is coupled by optical fibers to an 80 cm diameter integrating sphere. Open ports of the integrating sphere act as Lambertain light sources and so provide a diffuse illumination for the object under test. One or more cameras, placed in a front or side viewing position, capture images of the object

The *Chromasphere* system is our current reference device for in vivo skin color measurement and we have employed it in studies both internally and in our partner clinical research organizations (cf. ^15^ ^–^ ^18^). The Chromasphere system uses an 80 cm diameter integrating sphere combined with a fiber‐coupled light source to provide a diffuse illumination for color photography. In the standard configuration, the Chromasphere mounts three Hitachi HV‐F22F color cameras^19^ capturing evaluation images from a front, left‐side, and right‐side view of the face. We show a diagram of the Chromasphere system in Figure 1.

For more precise color measurements, we employ a Photo Research, Inc. PR‐650 SpectraScan spectroradiometer^20^ in the Chromasphere in place of the front‐view color camera. This instrument measures the average reflectance spectrum inside a 1° field of view (approximately 14 mm*φ* when used in the Chromasphere). This allows an analysis of object color independent from the scene illumination and free from metamerism or other color degeneracy effects, but does not provide any spatial resolution. The working spectral range of the PR‐650 is from 380 to 780 nm with a spectral resolution on the order of 4 nm and a bandwidth of 8 nm.

The HSI which we have developed to replace the HV‐F22F cameras is a spectral‐scanning instrument based around a Liquid Crystal Tunable Filter (LCTF). There are numerous technical considerations in the selection of a given approach to hyperspectral imaging and in the design of a particular device which we will not cover here. Readers who are interested in a more complete discussion of different hyperspectral imaging methods and instruments may find reviews such as^21^ useful. In our case, we selected a spectral‐scanning approach due to our need for reasonably fast imaging over a large field of view, and, as we previously reported the complete design and performance evaluation of this prototype in,^14^ here we will give only an overview of the instrument capabilities.

Our HSI has a selectable bandwidth of 32 nm, 18 nm, or 10 nm full width at half maximum (FWHM, measured at *λ *= 555 nm) with a continuously variable central wavelength from 420 to 730 nm at a tuning accuracy of ±FWHM*/*10 nm. It uses a monochromatic Charged‐Coupled Device (CCD) camera with 2016 × 2016 active pixels, integrated with an optical assembly of our own design to provide a field of view of ≈ 32 cm^2^ when used in the Chromasphere (working distance of 80 cm).

For calibration, we implement all the standard CCD characterizations (cf. ^22^, ^23^, ^24^, ^25^), including subtraction of the CCD dark counts from the raw data, adjustment for the non‐uniformity of the CCD response, and measurement of the absolute white response of the total system using a 99% reflectance standard to provide spectral calibration. We also fully characterized the linearity and noise performance of the CCD itself, and, combined with the calibration data, this allows us to convert the raw CCD count information into a spectral reflectance curve for each spatial pixel in the field of view.

We developed a custom‐data acquisition software in C++^26^ and QT^27^ to operate this instrument and capture hyperspectral images, or *datacubes*, which encode the spatio‐spectral information from the field of view into three‐dimensional voxels (two spatial and one spectral dimension). To construct a hyperspectral image, we tune the LCTF to one wavelength and capture one frame recording the number of counts in each pixel in the tuned bandpass, and combine this raw count data with the calibration information to determine the percentage reflectance for that bandpass. We repeat this for each wavelength in a defined sequence to assemble the full datacube.

Our standard exposure sequence, which we used for all measurements presented here, is 31 wavelengths between 420 and 730 nm at the 32 nm bandwidth setting. So constructed, each hyperspectral datacube contains the percentage of light reflected for each wavelength bin in the measured range (420 nm, 730 nm) for each image pixel in the field of view (2016 pixels^2^) for a total of 126 megavoxels.

We combined this instrument with a comprehensive hyperspectral image analysis framework written by us in Python 3.^28^ This software allows color and spectral calculations over the hyperspectral images, the definition of ROI within them, and the analysis of measurements across large sets of data. Table 1 gives a summary of the specifications of the HSI in a side‐by‐side comparison with the P‐650 spectroradiometer and the previous HV‐F22F system.

**TABLE 1:** Characteristics of the PR‐650 spectroradiometer,^20^ the HyperSpectral Imager (HSI),^14^ and the HV‐F22F color camera.^19^ Spectral bandwidth is the Full‐Width‐at‐Half Maximum (FWHM) of the bandpass. Spectral resolution is the spectral measurement step size (for the standard configuration in the case of the HSI). The imaging time of the HSI is for our default sequence covering the full spectral range

	**HV‐F22F**	**PR650**	**HSI**
Active pixels	1360×1024	N.A.	2016×2016
Field of view	19×14 cm^2^	1^◦^	32 cm^2^
Spatial resolution (*μ*m)	160	N.A.	160
Spectral resolution (nm)	N.A.	8	10
Spectral bandwidth (nm)	N.A.	8	10, 18, 32
Spectral accuracy (nm)	RGB	±2	± FWHM/10
Spectral range (nm)	400:700	80:780	420:730
Imaging time (s)	≈ 0.1	*<* 1	9.8
Signal‐to‐noise ratio	Unknown	100	≈ 47

### 2.1 Validation test design

In order to validate the end‐to‐end HSI system, we conducted an evaluation study on three foundation formulas applied to a test panel of 9 models. For each product, we captured a hyperspectral image of each of the 9 models before (*T*_0_) and immediately after (*T*_imm_) product application. Looking at the product effect introduces an extra source of variability compared to measuring bare skin color, and this may seem to be at odds with an instrument validation test. However, we previously tested the HSI instrument itself using test targets,^14^ and our goal in this study was to validate the HSI system as an evaluation platform and to show that this system can replace the older color‐cameras without loss of generality. To those ends, we chose to replicate as closely as possible a typical foundation screening test. In fact, the additional variability is a primary feature of this study, as it allows us to assess the impact of operational issues, such as ROI targeting, on the results.

To test the repeatability of the instrumental results, we applied each product twice, on different days. In addition, two different operators took the same data for each repetition in order to assess the reproducibility of the results. We also measured the spectrum of an area of the right cheek using a PR‐650 spectroradiometer during each repetition and at each time point. In this report, we will use the spectroradiometer measurements as a comparison point for the HSI repeatability and reproducibility results. We organized these 12 independent tests (3 formulas, 2 repetitions, 2 instruments) on 3 days over the course of 3 weeks.

As products in this test, we selected three make‐up foundations, each with a different level of coverage as evaluated by expert sensory evaluation, and, for models, we selected nine models with a higher density of spots from among our standard panelists. For each independent test, we applied the product and took measurements according to the test protocol shown in Table 2. To mimic a standard screening evaluation, we ranked the products in these tests according to the change in CIE *L* ^∗^ *a* ^∗^ *b* ^∗ 29^ values between before (*T*_0_) and immediately after (*T*_imm_) application, averaged over models in the test dataset. While the HSI and PR‐650 provide spectral information, we chose to work in terms of *L* ^∗^ *a* ^∗^ *b* ^∗^ values so that we could compare the HSI results against the legacy protocols, which means primarily the *L* ^∗^ *a* ^∗^ *b* ^∗^ results from the three CCD camera system.

**TABLE 2:** The protocol for this test. We applied the product under test according to steps listed in the table for each test repetition

1	Wash with cleansing oil and foaming cleanser (no moisturizer)
2	Moisturize with cosmetic water and milky lotion
3	Wait for 15 min
4	Perform *T*_◦_ measurement(s)
5	Product application by operator
6	Wait 10 min for product to dry
7	Perform *T*_imm_ measurement(s)

At each time point, we extract the average color value from a rectangular area on the right cheek of each model. For the PR‐650, the operator manually targets the ROI during acquisition, extracting the average spectrum from a region on the model's right cheek of approximately 14 mm diameter. For the HSI on the other hand, we extract the ROI during analysis by finding the location of 68 facial landmark points using the DLIB^30^ Histogram‐Orientation‐Gradient based face detector. We then define our ROI, 75 × 75 pixels^2^ on the right and left cheeks, by referencing these facial landmarks to build a hierarchy of ROI within each datacube. Table 3 contains a summary of the ROI used in this study by instrument.

**TABLE 3:** Definition of regions of interest (ROI) used for each instrument in this study. The PR‐650 is manually aimed during acquisition. For the HSI, we select the ROI automatically using face recognition and image segmentation during analysis

Instrument ROI name	Selection method	Size
HSI	Right cheek automatic face landmark recognition	12 × 12 mm^2^ (75 × 75 pixels^2^)
PR‐650	Right cheek manual aiming	≈ 14 mm *φ*

For both the HSI and PR‐650, we calculate *L* ^∗^ *a* ^∗^ *b* ^∗^ coordinates from the measured spectra for the CIE 1964 10° observer^31^, ^32^, ^33^ under D65 illumination,^34^ as we previously detailed in.^14^ We evaluated the statistical significance of any differences in these measured values between products using the Python Statsmodel^35^ library implementation of the Analysis of Variance (ANOVA) algorithm^36^ to determine if we can reject the null hypothesis (that no difference exists), followed by pairwise multiple comparison of means (Tukey HSD)^37^ to group the products into statistically distinct subsets if warranted. We used a test significance of 0.01 for both the ANOVA and the Tukey HSD tests.

This type of analysis is useful because our past foundation screening tests often reported their results as a relative ranking of the product effect on *L* ^∗^ *a* ^∗^ *b* ^∗^ values, along with an associated statistical analysis to determine if the observed difference between the product effects was credible. As a comparison between different instruments, we ideally expect that each gives the same relative ranking of the products in the test for each parameter. Any differences in the statistical grouping between instrument datasets can indicate differences in sensitivity between instruments or methods, or test variability for which we have not properly accounted. Discrepancies of the relative ranking between instruments or repetitions, on the other hand, can indicate at a glance some limitations or issues with the instruments or test design which we need to investigate.

## 3 RESULTS

The total validation dataset includes 216 hyperspectral images and 108 spectra obtained with the PR650. After completing all the data taking sessions, we construct the datacube from each hyperspectral image as we detailed in the previous section. Of these datacubes, we had to remove three from the first test day due to an error in the data acquisition software. As a result, the HSI dataset for the first repetition for the first operator contains fewer samples, and the complete time‐difference HSI data set contains 105 unique data‐points.

**FIGURE 2:**
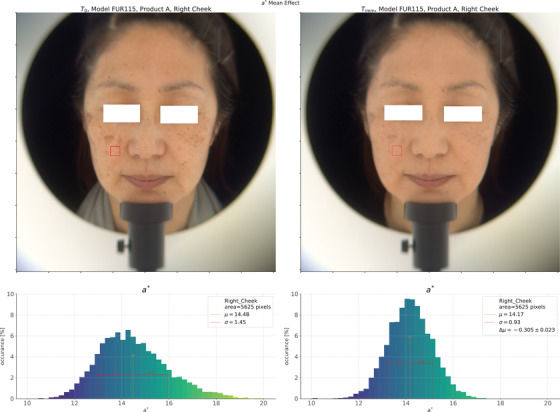
Example of results from the HyperSpectral Imager (HSI). Here, we show the model who was the closest to the mean effect in ∆*a* ^∗^ for product A in the ROI *Right Cheek* between the times *T*_0_, and *T*_imm_. We draw the regions of interest (ROI) on the color image reconstructed from the HSI spectral data (under D65 illumination) for each time‐point. The histograms show the distribution of *a* ^∗^ in the ROI, with the ROI mean value and standard deviation indicated

**FIGURE 3:**
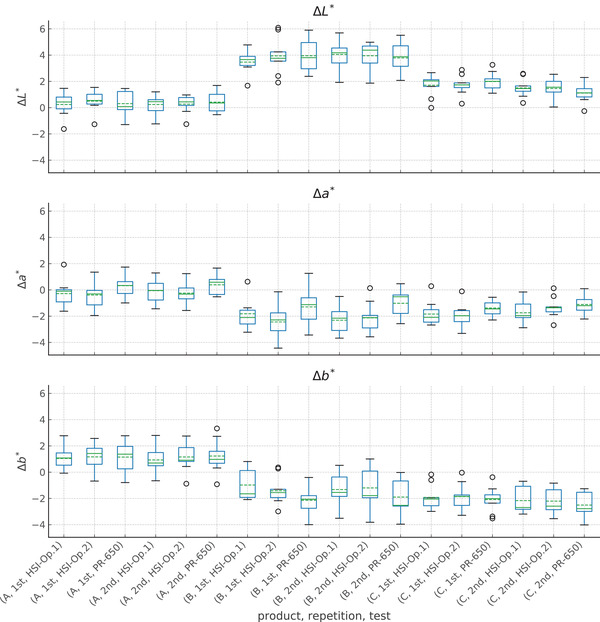
Box plot of ∆*L* ^∗^, ∆*a* ^∗^, and ∆*b* ^∗^. We plot the values by product, repetition, and test in abscissa, at the time ∆*T*_imm_, for the *Right_Cheek* ROI. The box extends from the first quartile (*Q*_1_) to the third quartile (*Q*_3_). The solid colored line in the box denotes the median value (*Q*_2_) of the set, while the dashed line shows the mean. The whiskers denote the range ±1.5(*Q*_3_ −*Q*_1_). Circles mark outliers, if any. We can observe that the three products seem to give a different effect for ∆*L* ^∗^, while there are two effect groups for ∆*a* ^∗^ and ∆*b* ^∗^. It also appears that the results roughly agree between the two instruments and the repetitions, although ∆*a* ^∗^ and ∆*b* ^∗^ for products B and C show variations which require closer inspection

We show an example image from this dataset in Figure 2. In the figure, we show an analysis of the *T*_0_ and *T*_imm_ images for the model closest to the *mean* change in ∆*a* ^∗^ for product B, shown with the working ROI outlined in red. We create these color images from the HSI spectral data by converting the *L* ^∗^ *a* ^∗^ *b* ^∗^ values to sRGB^38^ under D65 illumination and save the image in PNG format using the Open CV library.^39^ Below each image is a histogram of the *a* ^∗^ values in the ROI, with the two histograms on the same scale to facilitate comparison before and after product application. We also indicate the mean value of the parameter within the ROI and the change between the time points in the plot legend. In Figure 3, we show a box plot of the ∆*L*, ∆*a* ^∗^, and ∆*b* ^∗^ results across the full dataset, grouped by product, repetition, and operator. Here, we have included the PR‐650 data as a comparison point for the HSI results.

We give the results of statistical grouping analysis for the second repetition of the second operator in Table 4 as example of the analysis we performed for each operator–repetition–instrument set. Each entry in the *Ranking* column of the table lists the products in order of the magnitude of change, with the smallest (most negative) change first. A comma between product names indicates separate statistical groups, as determined by ANOVA analysis. For example, the entry “AC,B” for the ∆*L* results (first row of Table 4) indicates that product A showed the smallest change in *L* ^∗^ and product B showed the greatest. The comma between products C and B indicates that the difference in ∆*L* between those products is statistically significant, while the effect on L^∗^ of products A and C is not statistical distinct within our chosen 1% significance threshold.

**TABLE 4:** Statistical analysis of products grouped by *L* ^∗^, *a* ^∗^, and *b* ^∗^ for the second repetition of the “HSI‐Op.2″ for ∆*T*_imm_. For each parameter, we show the ANOVA *p*‐value results, as well as the multiple comparison of means grouping analysis. For each parameter, we list the product with the smallest (most negative) value first and commas separate the statistical groups. We give the mean value for each product in the Results column in order of the grouping, with parentheses to indicate groups. In this particular sub‐test, we find two subgroups for ∆*L* ^∗^ and ∆*a* ^∗^ effect, and three groups for ∆*b* ^∗^ effect. For all three parameters, the ANOVA *p*‐value is well below 0.01 indicating a reasonably low probability of observing these results in the null hypothesis case

**Parameter**	***N*_Groups_**	**Ranking**	**Results**	***p***
∆*L* ^∗^	2	AC, B	(0.27, 1.46), 3.95	9.2·10^−9^
∆*a* ^∗^	2	B, CA	−2.13, (−1.34, − 0.24)	1.3·10^−3^
∆*b* ^∗^	3	C, B, A	−2.2, −1.2, 1.16	2.1·10^−5^

We summarize the grouping results from all tests and repetitions in Table 5. As a further example from that table, the entry “CBA” for the ∆*a* ^∗^ result of the first repetition of HSI‐Op.1 (first row, fifth column of Table 5) indicates that product C gave the smallest (most negative) change in *a* ^∗^ and product A the largest, but with all products occupying the same statistical group (i.e., no significance difference between product effects). As previously mentioned, this grouping analysis is useful for quickly understanding if the results of each instrument–operator–repetition instance are consistent. If the results from all instruments, operators, and repetitions are equivalent, then for each column in Table 5 all rows should be the same. We will discuss the actual results in the next section. We also give a summary of all numerical results in Table 6.

**TABLE 5:** Summary of product rankings for each attribute across instruments, operators, tests, and repetitions. For each entry, we list the product with the smallest (most negative) value first. Commas separate statistical groups as found with multiple comparison of means. For example, “AC,B” indicates that A has the smallest value and B the greatest, with A and C occupying the same statistical group. The ranking of products by ∆*L* is consistent across all sub‐tests, although there are some differences between the statistical groups. For ∆*a* ^∗^ and ∆*b* ^∗^, the groups are consistent between all HSI sub‐tests (outside that of ∆*a* ^∗^ for the first repetition of operator Op.1, which had a smaller sample size). However, there is some inconsistency between the HSI and PR‐650 results for these values, as well as between repetitions of the PR‐650. The ∆*E* results are consistent across all sub‐tests. See the text for further discussion

**Instrument repetition test**		∆*L* ^*^	**∆*a* ^∗^**	**∆*b* ^∗^**	**∆*E***
HSI	1st	Op.1 A,C,B	CBA	CBA	AC,B
	2nd	Op.1 AC,B	B,C,A	C,B,A	AC,B
HSI	1st	Op.2 AC,B	B,C,A	C,B,A	AC,B
	2nd	Op.2 AC,B	B,C,A	C,B,A	AC,B
PR‐650	1st	– A,C,B	C,B,A	B,C,A	AC,B
	2nd	– AC,B	C,B,A	C,B,A	AC,B

**TABLE 6:** Summary of ∆*T*_imm_ (change relative to *T*_0_) results for the Right_Cheek ROI for each product, repetition, and test. We report each value as the mean effect ± the standard deviation of the effect over the sample set

**Product repetition test**			**∆*L* ^∗^**	**∆*a* ^∗^**	**∆*b* ^∗^**
A	1st	HSI‐Op.1	0.24 ± 0.93	−0.28 ± 1.06	1.04 ± 0.94
		HSI‐Op.2	0.50 ± 0.80	−0.39 ± 0.97	1.17 ± 1.07
		PR‐650	0.31 ± 0.93	0.32 ± 0.81	1.15 ± 1.18
	2nd	HSI‐Op.1	0.24 ± 0.76	−0.06 ± 0.90	0.93 ± 1.21
		HSI‐Op.2	0.27 ± 0.69	−0.24 ± 0.84	1.16 ± 1.12
		PR‐650	0.42 ± 0.78	0.39 ± 0.80	1.23 ± 1.26
B	1st	HSI‐Op.1	3.47 ± 1.04	−1.82 ± 1.36	−0.98 ± 1.34
		HSI‐Op.2	3.94 ± 1.39	−2.44 ± 1.24	−1.41 ± 1.09
		PR‐650	3.95 ± 1.22	−1.29 ± 1.40	−2.14 ± 1.08
	2nd	HSI‐Op.1	4.05 ± 1.17	−2.31 ± 1.00	−1.32 ± 1.34
		HSI‐Op.2	3.95 ± 1.04	−2.13 ± 1.17	−1.20 ± 1.54
		PR‐650	3.79 ± 1.21	−1.02 ± 1.00	−1.89 ± 1.30
C	1st	HSI‐Op.1	1.71 ± 0.88	−1.84 ± 0.95	−1.92 ± 0.94
		HSI‐Op.2	1.73±0.74	−1.96 ± 0.89	−1.86 ± 0.99
		PR‐650	1.99 ± 0.70	−1.38 ± 0.55	−2.08 ± 0.97
	2nd	HSI‐Op.1	1.51 ± 0.71	−1.74 ± 0.88	−2.17 ± 1.00
		HSI‐Op.2	1.46 ± 0.77	−1.34 ± 0.80	−2.20 ± 0.98
		PR‐650	1.11 ± 0.72	−1.11 ± 0.71	−2.50 ± 0.95

In addition to a comparison of the study results between sub‐tests, we also compared each independent measurement between repetitions and operators for each instrument. For each model, product, time point, and repetition, we plot the measurement from one HSI operator versus the other in Figure 5. After plotting, we perform a linear least‐squares regression to assess the relationship between the two datasets. We also report the percentage nonlinearity between ordinate measurements and the fit result, which we define as

(1) $$\%\mathrm{NL}=100\frac{\;\delta_{+}+\left|\delta_{-}\right|}{\mathrm{Maximum}\;\mathrm{Signal}}$$

where *δ*_+_ and *δ*_−_ are the maximum positive and negative differences between the linear least‐squares regression and the measured value over the range. Finally, we report the average difference between the measurements from each instrument, ∆. Where possible, we indicate the product and repetition subgroups on each plot for reference, but we do all correlation analyses over the full dataset. In Figures 6 and 7 we show the correlation between repetitions for the HSI and the PR‐650 data. In both cases, we do the same analysis as for the correlation between operators, and we indicate the test (operator) and product subgroups on each plot for reference.

## 4 DISCUSSION

**FIGURE 4:**
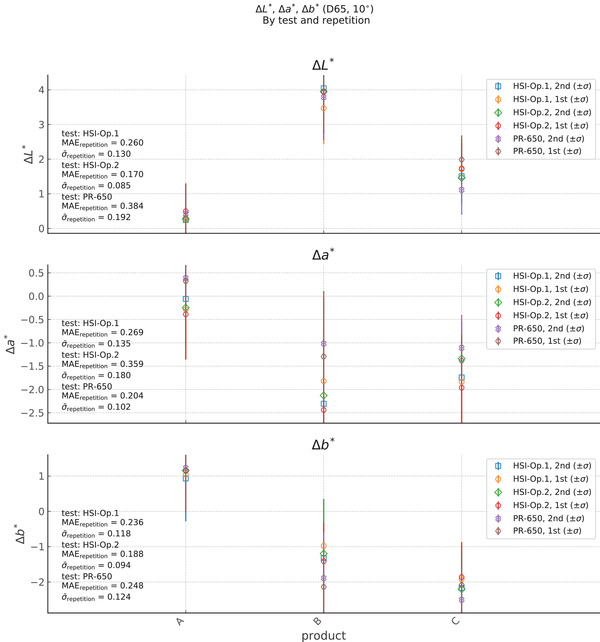
Plot of ∆*L* ^∗^, ∆*a* ^∗^, and ∆*b* ^∗^. Here, we plot the measured values with the product in abscissa for each test and repetition in the data set, at the time ∆*T*_imm_, coming from the *Right_Cheek* ROI. The error bars indicate the standard deviation over the 9‐model sample set. We can see here that the overall results appear to agree between the sub‐tests, although there are distinct differences in the results from the PR‐650 and the HyperSpectral Imager (HSI) for ∆*a* ^∗^, and ∆*b* ^∗^, especially in the case of products B and C

We showed a boxplot of the all results grouped by product, test, and repetition in Figure 4. This type of box plot allows for a quick visual comparison of the results across groups, including outliers and their effect on the set means and medians. Here, we also complement the boxplot presentation with a standard plot of the mean result by product for each test in Figure 4, and there we also show the mean average error (MAE) between the results for relevant repetition and test pairs.

From those plots and Table 5, we find that the ranking of products by ∆*L* is the same across all subtests, although there are some differences between the statistical groups for the first repetition of *HSI.Op1* and the PR‐650. For ∆*a* ^∗^ and ∆*b* ^∗^, the groups are consistent between all HSI sub‐tests, aside from the ∆*a* ^∗^ results for the first repetition of *HSI.Op1*. As we mentioned in the dataset summary, however, that sub‐test had fewer samples (6 as opposed to 9) due to an error in the data acquisition software, and this accounts for the difference in the results, as the magnitude of the difference between products C and B is small and missing one third of the samples can easily shift the mean result.

If we compare the HSI results with those from the PR‐650, we find that the spectroradiometer does not consistently agree with the HSI results for ∆*a* ^∗^ and ∆*b* ^∗^. In particular, the results for product B and C change ordering (C,B) compared to those of the HSI (B,C). Looking at the results for these products in Table 6, however, we see that the difference between the product B and C effects are on the order of 0.1 (units of *b* ^∗^), which is close to the statistical uncertainty.

Furthermore, from Figure 4 we see that the PR‐650 gave a consistently smaller change in *a* ^∗^ and a consistently greater change in *b* ^∗^ than the HSI for products B and C. The spectroradiometer also gave a slight *increase* in *a* ^∗^ for product A, where the HSI reported a slight *decrease*. This points toward a difference in effect between ROI, that is, the exact result depends on where you look and is in effect a source of systematic uncertainty in the analysis. At the same time, there is also inconsistency in the grouping results for ∆*b* ^∗^ between the two repetitions of the PR‐650, which does not occur for the HSI outside the first repetition of the first operator where the total sample size is a third smaller. We intend to address the comparison of results between instruments in a separate paper, including a complete evaluation of the instrument uncertainties. These are important topics warranting detailed discussion, and we will limit the scope of this report to the repeatability and reproducibility of the HSI.

### 4.1 Reproducibility

**FIGURE 5:**
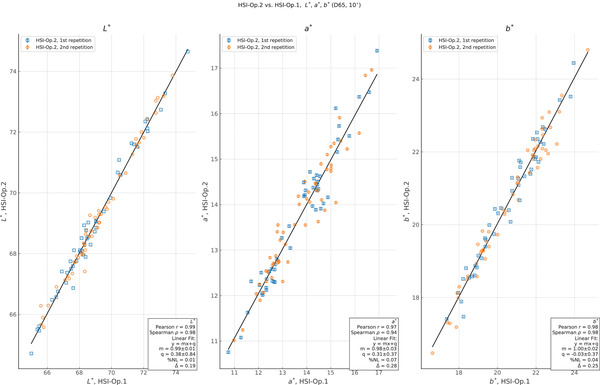
Plot of measurement correlation between tests for *L* ^∗^, *a* ^∗^, and *b* ^∗^ calculated for D65 (10°) illumination. Values are those measured during the test HyperSpectral Imager (HSI)‐Op.2 in ordinate vs. the HSI‐Op.1 in abscissa. For each value, we plot the estimated uncertainty of the ordinate measurement, although in some cases the error bar is similar in size to the marker. We show the results of a least‐squares linear fit, along with estimates of the Pearson *r* and Spearman *ρ* values for each quantity. The correlation of measurements between operators is good with the data showing a linear and monotone relationship

Looking at the reproducibility between operators for the HSI in Figure 5, we find a strong linear relationship between the two sets of results with *r *= (0.99, 0.97, 0.98) for *L* ^∗^, *a* ^∗^, and *b* ^∗^. The monotonicity is also good with *ρ *= (0.98, 0.94, 0.98). This indicates that the relative ranking of the results is well preserved between different HSI measurements, which is a key criteria for any relative evaluation measurements. In addition, the scale is consistent with 1.0 and the offset is consistent with 0 for all three parameters, and the average differences between measurement pairs are ∆ = (0.19, 0.28, 0.25) for *L* ^∗^, *a* ^∗^, and *b* ^∗^.

The statistical uncertainty of the HSI spectral measurements is approximately 2%. Propagating this uncertainty through the *L* ^∗^ *a* ^∗^ *b* ^∗^ equations, we find typical uncertainties (the average uncertainties from this dataset) on the pixel‐by‐pixel values of *σ_L_* ∗ = 0.3, *σ_a_* ∗ = 2.2, and *σ_b_* ∗ = 0.9 for skin tone colors under D65 illumination. As we take the average color value over a two‐dimensional ROI, the statistical uncertainty on the *average* color value in the ROI reduces in proportion to the square root of the number of pixels in the ROI, and the uncertainties on the difference of the color values add in quadrature, assuming that they are not correlated. For the 75 pixel^2^ ROI we used for the HSI in this test, we therefore expect the uncertainties on the difference of the *L* ^∗^ *a* ^∗^ *b* ^∗^ values before and after product application, averaged over each ROI, to be on the order of *σ*_∆_ *_L_* ∗ ≈ 0.01, *σ*_∆_ *_a_* ∗ ≈ 0.04, and *σ*_∆_ *_b_* ∗ ≈ 0.02.

If we look at the average results for each product in Figure 4, we see that the standard deviation between the two datasets is on the order of 0.05– 0.08 for the measured results of ∆*L* ^∗^, ∆*a* ^∗^, and ∆*b* ^∗^. We also find that the MAE between the two operators ranges from 0.1 to 0.15. The difference between the measurements taken by each operator is at the same order of magnitude as the instrument statistical uncertainty.

In view of this, the reproducibility results are consistent with the statistical uncertainty of the HSI measurements, combined with shifts in the model's positioning between operators leading to changes in viewing angle and ROI selection. Based on the technical analysis of the instrument calibration as given in ^14^, we believe that only the statistical uncertainty and the systematic uncertainty from the selection of a single ROI for the analysis limit the reproducibility of this instrument.

### 4.2 Repeatability

**FIGURE 6:**
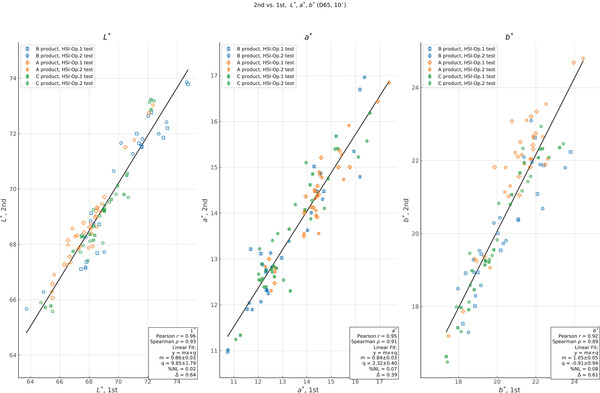
Plot of measurement correlation between repetitions for *L* ^∗^, *a* ^∗^, and *b* ^∗^ under D65 (10°) illumination. Values are those measured for the second repetition of the Hyperspectral Imager in ordinate vs. the first in abscissa. For each value, we plot the estimated uncertainty of the ordinate measurement. We show the results of a least‐squares linear fit, along with estimates of the Pearson *r* and Spearman *ρ* values for each quantity. Although this relationship is less linear than the reproducibility, these results nonetheless show the good repeatability of the HyperSpectral Imager (HSI) measurements

We showed a correlation plot between the two repetitions of the HSI in Figure 6. Here, the linearity and monotonicity between the repetitions are good, with *r *= (0.96, 0.95, 0.92) and *ρ *= (0.93, 0.91, 0.89) for *L* ^∗^, *a* ^∗^, and *b* ^∗^. The scale is consistent with 1.0 for *b* ^∗^ and the offset is consistent with 0 for this parameter. Both *L* ^∗^ and *a* ^∗^ show a scale factor less than 1 and a positive offset. At the same time, the average differences are ∆ = (0.64, 0.39, 0.61) for *L* ^∗^, *a* ^∗^, and *b* ^∗^, respectively. We find that the standard deviation between the two HSI repetitions is on the order of *σ *= 0.1–0.2, with MAEs in the range of 0.17–0.36 (see Figure 4).

**FIGURE 7:**
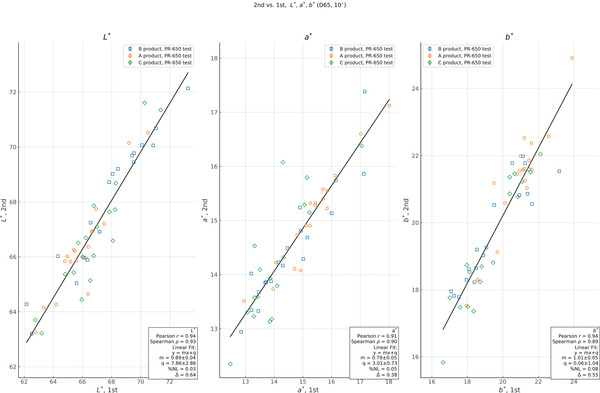
Plot of measurement correlation between repetitions for *L* ^∗^, *a* ^∗^, and *b* ^∗^, calculated for D65 (10°) illumination. Values are those measured for the first repetition of the PR‐650 Spectroradiometer in ordinate vs. the second in abscissa, not including any estimate of the instrumental uncertainty. We show the results of a least‐squares linear fit, along with estimates of the Pearson *r* and Spearman *ρ* values for each quantity

In Figure 7, we show the same correlation between the two repetitions of the PR‐650 measurements. Here, the linearity and monotonicity between the repetitions are again good, with *r *= (0.94, 0.91, 0.94) and *ρ *= (0.93, 0.90, 0.89) for *L* ^∗^, *a* ^∗^, and *b* ^∗^, although the relationship is less linear or monotone than for the HSI repetitions. The scale of the *b* ^∗^ relationship is consistent with 1.0 with zero offset, while the scale factors of *L* ^∗^ and *a* ^∗^ are less than 1 and these parameters both have a positive offset. The average differences between the measurements of the two repetitions are ∆ = (0.64, 0.38, 0.55) for *L* ^∗^, *a* ^∗^, and *b* ^∗^, respectively. From Figure 4, we find that the standard deviation between the two repetitions is on the order of *σ *= 0.1–0.2, with MAEs in the range of 0.17–0.36. Based on these results, we conclude that the HSI shows a better repeatability than the PR‐650. That the HSI should be better is also in line with benefits afforded by the HSI's data handling and analysis software. In particular, the PR‐650 suffers in terms of repeatability due to the need to manually target the ROI during acquisition.

It is also important to remember that the repeatability of these tests is the combination of the repeatability of the instrument's measurements, the test protocol, and the actual product effect. This effectively means that the repeatability is the convolution of the instrumental statistical (count noise) and systematic (ROI selection) uncertainties, with the uncertainty introduced by test protocol steps, such as the amount of makeup applied, and any actual changes in product effect between repetitions. The latter is not unlikely in this test design as we applied a revolving line of products to the same nine models over the space of 3 weeks, and it is not difficult to imagine that there were shifts in the models bare skin color over this time. Any such changes in bare skin color can give a corresponding change in product effect and result in a non‐linear change in the measured color difference between repetitions.

From a purely technical perspective, we have no reason to expect that the measurements from the HSI will vary from one test day to another as we perform a daily calibration routine which takes account of variations in both the illumination and instrument over time. Furthermore, considering the good *reproducibility* of the HSI results, the difference between the repetitions, beyond the lower limit set by the reproducibility, likely represents the repeatability of the test protocol itself combined with an actual divergence in the product effect. The presence of some number of outliers in the correlation plots where there was a large different between repetitions, and indications of correlations in the overall results from each instrument between repetitions seem to support the latter.

## 5 CONCLUSION

We have developed a novel HSI able of taking full‐face format images as a next‐generation platform for the evaluation of skin color in vivo. In order to validate the capabilities of this system, we replicated a typical make‐up foundation screening test by applying a selection of three products in turn to a panel of nine models. We repeated this test twice in order to study the repeatability of the HSI results, and during each test had all measurements duplicated by two different operators to assess the instrument's reproducibility.

Using the HSI measurements, we find that the product ranking by change in *L* ^∗^, *a* ^∗^, *b* ^∗^, and ∆*E* are consistent over multiple repetitions of the same test and between different operators. In terms of measurement reproducibility, we find that the HSI performs well, and the signal‐to‐noise ratio of the instrument and systematic uncertainties due to the selection of and analysis through a single working ROI are the primary sources of non‐reproducibility. This leads us to believe that it is fundamental to study the systematic uncertainty due to ROI selection and develop new techniques to address this question.

Regarding measurement repeatability, we likewise find that the HSI gives highly repeatable results, outperforming the PR‐650 which was our previous standard for precision color measurement. In this case, the main sources of test non‐repeatability appear to come from the limit of the instrument reproducibility, variance coming from the test design, protocol, and analysis‐method, and actual variations in product effect between tests. Operationally, we found in these tests that the HSI gives clear advantages over either the PR‐650 or a standard color camera alone, as it provides a true spectral measurement of the color combined with good spatial resolution. This is particularly true given that any measurement result may depend on where you look, and as the PR‐650 is manually aimed it is difficult to study this uncertainty. The *post hoc* selection of the study ROI possible with the HSI, on the other hand, allows us to properly address the question of ROI‐related systematic uncertainties, which are one of the main limits on the measurement reproducibility and repeatability.

Compared to a typical color camera on the other hand, the fact that the HSI provides a spectral measurement allows for analysis of color under different illuminations and other analyses using the full spectral information in the scene which are not possible with RGB imaging. Beyond that, a comparison between instruments over the test repetitions, which we have not reported in detail here, raises interesting points about the interpretation of evaluation study results, and the need to properly account for instrumental uncertainties in study analyses. We intend to address the comparison of results of these studies between instruments and the issue of instrument uncertainties in a separate paper.

Based on the repeatability and reproducibility results of this study, we conclude that the HSI performs well in color evaluation in place of both the PR‐650 spectroradiometer and the HV‐F22F color camera. While the starting goal of the HSI project was to create a next‐generation platform for the color evaluation of cosmetic products, hyperspectral imaging goes beyond color and gives us full access to the range of analysis options made possible by characterization of both an object's spectrum and the change in spectrum with location. Based on that, we foresee a range of future analysis methods which we can leverage for evaluation of skin condition and product effect, and the results of this validation show that those future studies and methods will rest on a strong instrumental foundation.

## CONFLICT OF INTEREST

The authors declare that there is no conflict of interest that could be perceived as prejudicing the impartiality of the research reported.
